# Serum 25 hydroxyvitamin D status in 6‐month‐old infants in Guangzhou, China: A paired longitudinal follow up study

**DOI:** 10.1111/mcn.12924

**Published:** 2020-01-22

**Authors:** Jing Wang, Joy Yue Zhang, Ru Wei, Shunping Hu, Tao Lin, Gendie E. Lash, Meizhen Tan

**Affiliations:** ^1^ Department of Children Health Care, Guangzhou Institute of Pediatrics, Guangzhou Women and Children's Medical Center Guangzhou Medical University Guangzhou China; ^2^ Division of Uterine Vascular Biology, Guangzhou Institute of Pediatrics, Guangzhou Women and Children's Medical Center Guangzhou Medical University Guangzhou China; ^3^ Department of Obstetrics, Guangzhou Institute of Pediatrics, Guangzhou Women and Children's Medical Center Guangzhou Medical University Guangzhou China; ^4^ Virus Laboratory, Guangzhou Institute of Pediatrics, Guangzhou Women and Children's Medical Center Guangzhou Medical University Guangzhou China

**Keywords:** 6‐month infant, 25‐hydroxyvitamin D, feeding patterns, parental education, season, supplementation

## Abstract

To assess the vitamin D status in healthy 6‐month‐old infants, as well as vitamin D supplementation and feeding patterns in Guangzhou, China, serum 25‐hydroxyvitamin D (25OHD) concentrations of 202 infants were measured at birth (cord blood) and at 6 months of age in Guangzhou, China. Questionnaires acquiring demographic characteristics, maternal and infantile vitamin D supplementation during pregnancy and first 6 months after birth, and feeding patterns during the first 6 months were completed by participating mothers. Physical examinations and blood sampling were carried out among infants at 6 months of age. The majority of infants (93.6%) were supplemented with vitamin D during the first 6 months of life on a voluntary basis. The *M* ± *SD* of cord serum 25OHD concentration was 46.2 ± 16.4 nmol/L, whereas the *M* ± *SD* of 25OHD concentration at 6 months was 82.9 ± 24.9 nmol/L. Serum 25OHD concentrations <30 nmol/L were seen in 34 (16.8%) infants at birth but only one (0.5%) at 6 months. Only 11 (5.4%) infants had concentrations >75 nmol/L at birth, whereas the majority of infants (*n* = 131, 64.9%) had concentrations >75 nmol/L at 6 months. The main predictors of 25OHD levels at 6 months included season, vitamin D supplementation, parental education level, and feeding patterns. To conclude, serum 25OHD concentrations were low at birth in a southern Chinese population, and infantile supplementation is an effective way to improve 25OHD status. Exclusively breastfed infants might need greater vitamin D supplementation, and individualized vitamin D supplementation plans might be needed.

**Key messages**

- Vitamin D deficiency is widespread in new born infants in China.
- Infants' vitamin D status at 6 months is much improved by vitamin D supplementation.
- The current Chinese vitamin D supplementation guidelines do not meet the needs of one third of infants at 6 months.
- The main predictors of 25OHD levels at 6 months included season, vitamin D supplementation, parental education level, and feeding patterns.
- An individualized vitamin D supplementation plan might be needed in the future.

## 1 INTRODUCTION

Vitamin D plays an important role in growth and development during early life, not only for its role in bone development (Moreno, Valtuena, Perez‐Lopez, & Gonzalez‐Gross, 2011; Turner, Anderson, & Morris, 2012). The main source of vitamin D is through skin synthesis under exposure to ultraviolet B‐rays light (wavelength 290–315 nm; Hollis & Wagner, 2004). There are limited food sources that naturally contain vitamin D, mainly oily ocean fish, egg yolk that contains vitamin D_3_, ultraviolet‐radiated mushrooms that contain vitamin D_2_ and fortified dairy products. Nutritional supplementation of vitamin D is an important way to prevent vitamin D deficiency not only in infancy but for the whole life span, particularly in a population that eschews exposure to the sun, such as covering up with clothing and use of sunshade umbrellas, hats, and sunscreen. Human milk contains very little vitamin D (Streym et al., 2016), and is insufficient to meet the nutritional requirement of infants during early life if the mother is vitamin D deficient (Thiele, Senti, & Anderson, 2013). Therefore, vitamin D supplementation is crucial, particularly for breastfed infants. Guidelines for vitamin D supplementation in infants differ from country to country and have been modified several times during the past decade (Mimouni & Shamir, 2009). Since 2008, the Chinese Medical Association recommends that all children (including infants) must receive 400 IU/day of vitamin D until the age of 2 years (*Chinese Journal of Pediatrics.,* 2008), although studies showed that vitamin D deficiency may develop even in supplemented infants (Greer, 2008; Onal, Adal, Alpaslan, Ersen, & Aydin, 2010). This study was designed to assess the vitamin D status in healthy Chinese infants at birth and 6 months taking into consideration vitamin D supplementation, season of sampling and different feeding patterns in Guangzhou.

## 2 METHOD

### 2.1 Study design

**Figure 1:**
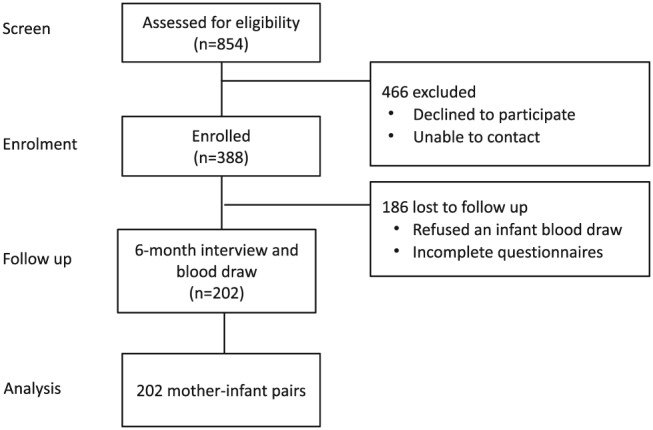
Flow chart of the participants in the study

A total of 854 mothers were recruited to take part in a birth cohort. Inclusion criteria were apparently normal pregnancy, singleton birth, and vaginal delivery. Written informed consent was obtained from all mothers prior to collection of umbilical cord blood samples. Only 388 (45.4%) returned to the hospital after giving birth when called for interview. All the subjects in this study were of Han ethnicity. Additional reasons for loss of follow‐up were mothers refused an infant blood draw (*n* = 167, 43.0%) and incomplete questionnaires (*n* = 19, 4.9%). The final number remaining in this study was 202 infants (Figure 1). Questionnaires obtaining information including demographic information, vitamin D supplementation, and feeding patterns were carried out at 6 month visits. Physical examinations including body weight, height, and head circumference were also performed. Blood samples were taken and kept at 4°C until centrifuged (15 min at 3,000 g) and serum stored at −80°C until required for analysis.

This study was conducted according to the Declaration of Helsinki guidelines, and all procedures were approved by Guangzhou Women and Children's Medical Center (GWCMC) Ethical Review Board.

### 2.2 Analytical methods

Total serum 25‐hydroxyvitamin D (25OHD_2_ plus 25OHD_3_) was determined using enzyme immunoassay (Immuno Diagnostic System, AC‐57SF1, Boldon, UK) in the Virus Laboratory, Guangzhou Institute of Pediatrics, GWCMC. The inter‐ and intra‐assay coefficients of variation were both below 5%. Quality control analysis was carried out on a daily basis.

### 2.3 Statistical analysis

Statistical analysis was conducted using PASW Statistics Version 20.0 (SPSS, IBM, USA). The distribution of all variables was tested by Kolmogorov–Smirnov. Descriptive statistics (mean and standard deviation (*SD*), median and interquartile range, and frequency and percentage) were determined for all variables. Primary correlations were explored using Pearson's test (parametric) and Spearman's test (non‐parametric) depending on data distribution. The serum 25OHD concentrations at both birth and 6 months were slightly deviated from the normal distribution; therefore, square‐root transformation was used, and the residuals were approximately normally distributed. Differences between two groups were tested using Student's *t* test and between three or more groups using analysis of variance and using both original and transformed values. In addition, non‐parametric tests were performed on the original values. Parametric tests using either transformed and nontransformed values or non‐parametric tests using original values generated similar results; therefore, parametric tests using transformed values were reported. The regression models on the original values and the square‐root transformed values identified the same factors as being significantly associated with 25OHD; therefore, we have reported the results from the linear regression model without transformation. A two‐sided test with a *p* value <.05 was considered statistically significant.

## 3 RESULTS

### 3.1 25OHD status at birth and 6 months

**Figure 2:**
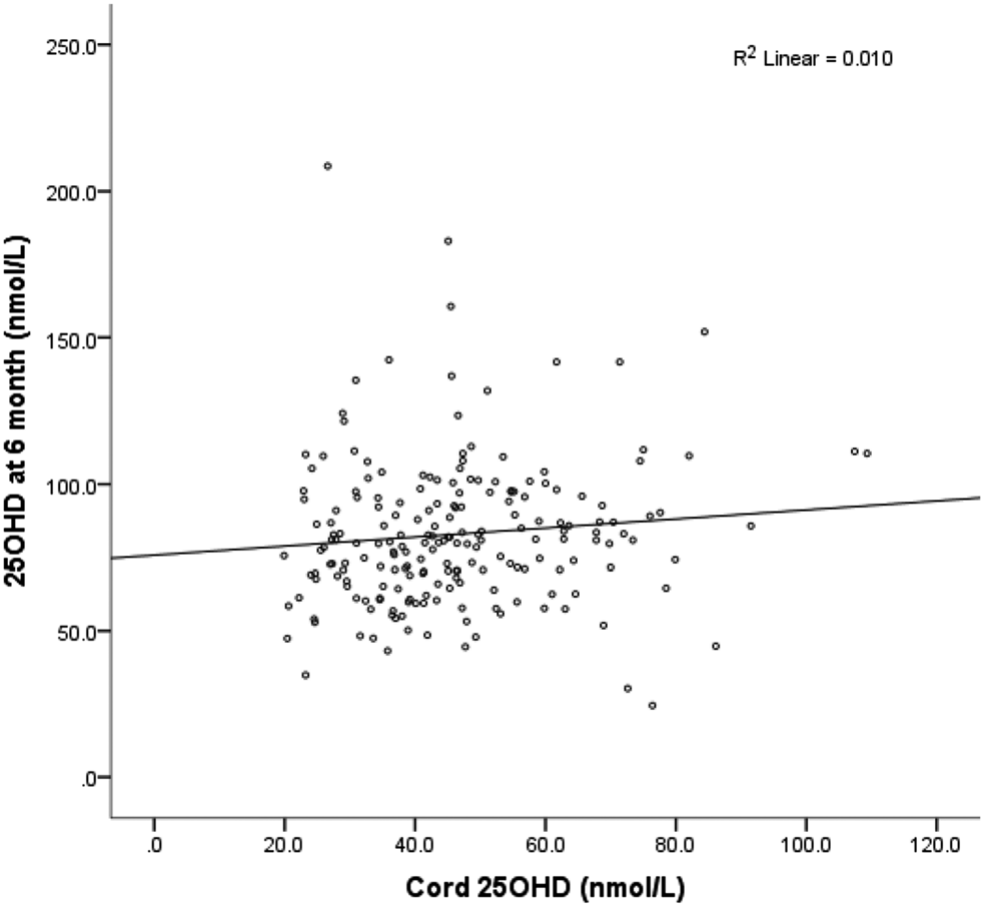
Cord blood 25OHD and 25OHD at 6 months in 202 infants born in Guangzhou, China. 25OHD, 25‐hydroxyvitamin D

The principle characteristics including parental information of the subjects enrolled in the study are displayed in Table 1. There was no difference in the principle characteristics of parents and infants included in the study and those lost to follow up (Table 2). The majority of infants (93.6%) were supplemented with vitamin D during the first 6 months of life on a voluntary basis. The *M* ± *SD* (nmol/L) concentration of cord serum 25OHD at birth was 46.2 ± 16.4 with median (interquartile range **[**IQR]) 44.7 (34.6, 55.2) and ranging from 19.9 to 109.3 nmol/L. Cord serum 25OHD concentrations <30 nmol/L were seen in 34 (16.8%) infants, and concentrations between 30 and 50 nmol/L were reported in 102 (50.5%) infants, and only a minority of infants (*n* = 11, 5.4%) had concentrations >75 nmol/L. At the age of 6 months, the serum 25OHD concentrations had nearly doubled with *M* ± *SD* concentrations of 82.9 ± 24.9 nmol/L with median (IQR) of 80.8 (67.5, 95.7) and ranging from 24.9 to 208.6 nmol/L. The raw mean difference of serum 25OHD between cord and at 6 months was 36.7 ± 2.1 nmol/L. Severe vitamin D deficiency (serum 25OHD concentrations <30 nmol/L) was nearly depleted at 6 months and was only seen in one participant. A small proportion of infants (*n* = 10, 5.0%) had 25OHD between 30 and 50 nmol/L, and the majority of infants (*n* = 120, 59.4%) had concentrations >75 nmol/L at 6 months. Cord serum 25OHD concentrations were positively related with the concentration at 6 months (*r* = 0.149, *p* = .03; Figure 2), but not to a great extent likely due to the season in which the measurements were taken.

**Table 1:** Principle parental and infant characteristics of the subjects enrolled in the study (*n* = 202)

Variable	Measure
Maternal childbearing age (year)	30.3 (27.7, 33.6)
Paternal childbearing age (year)	31.4 (28.8, 35.7)
Maternal college education (%)	73.8
Paternal college education (%)	69.8
Maternal use of vitamin D supplements during pregnancy (%)	89.1
Maternal vitamin D supplements during pregnancy (IU/day)	500 (200, 833)
Maternal use of vitamin D supplements during lactation (%)	58.6
Maternal vitamin D supplements during lactation (IU/day)	500 (200, 833)
Infant sex—male (%)	60.9
Infant birth weight (kg)	3.3 ± 0.3
Infant birth length (cm)	50 (49, 51)
Infant age at clinical visit (days)	180 (178, 181)
Infant weight at 6 months (kg)	7.8 (7.3, 8.4)
Infant height at 6 months (cm)	67.3 ± 2.3
Infant head circumference at 6 months (cm)	42.4 ± 1.2
Exclusively breastfed (%)	54.0
Initiated weaning at 6 months (%)	81.7
Infants supplemented with vitamin D at 6 months (%)	93.6
Cord blood 25OHD (nmol/L)	44.7 (34.6, 55.2)
Serum 25OHD at 6 months (nmol/L)	80.8 (67.5, 95.7)

*Note*. *M* ± *SD* (all such values) and median; interquartile range in parentheses (all such values); used in the case of non‐normally distributed variables.

Abbreviation: 25OHD, 25‐hydroxyvitamin D.

**Table 2:** Principle characteristics of parents and infants between the patients who completed the study or were lost to follow up

Variable	Completed the study (*n* = 202)	Lost to follow up (*n* = 186)	*p* value
Maternal childbearing age (year)	30.3 (27.7, 33.6)	30.5 (27.8, 34.0)	.9
Paternal childbearing age (year)	31.4 (28.8, 35.7)	32.7 (29.4, 36.3)	.06
Maternal college education (%)	74.0	77.5	.6
Paternal college education (%)	69.6	78.9	.1
Maternal use of vitamin D supplements during pregnancy (%)	89.1	83.3	.1
Infant birth weight (kg)	3.3 ± 0.3	3.2 ± 0.4	.2
Infant birth length (cm)	50 (49, 51)	50 (49, 51)	.3
Infant age at clinical visit (days)	50 (49, 51)	50 (49, 51)	.6
Infant weight at 6 months (kg)	7.8 (7.3, 8.4)	7.8 (7.3, 8.4)	.7
Infant height at 6 months (cm)	67.3 ± 2.3	67.3 ± 2.4	.9
Infant head circumference at 6 months (cm)	42.4 ± 1.2	42.5 ± 1.2	.8
Exclusively breastfed (%)	54.0	54.9	.9
Initiated weaning at 6 months (%)	81.9	76.6	.4
Infants supplemented with vitamin D at 6 months (%)	93.6	91.9	.6
Cord blood 25OHD (nmol/L)	44.7 (34.6, 55.2)	43.2 (29.4, 57.7)	.5

*Note. M* ± *SD* (all such values) and median; interquartile range in parentheses (all such values); used in the case of non‐normally distributed variables. Differences between two groups were tested using analysis of independent *t* test for normally distributed variables and independent *U* test for non‐normally distributed variables. The chi‐square test was used to compare the rates.

Abbreviation: 25OHD, 25‐hydroxyvitamin D.

### 3.2 Determinates of 25OHD at 6 months

In this study, we found infants born to higher educated parents (both paternal and maternal), summer season of blood sampling (May–October), and with vitamin D supplementation had higher 25OHD concentrations at 6 months (Table 2). Infants born to parents who both had a college education had the highest 25OHD concentrations at 6 months, and the mean difference between both college educated parents and couples where at least one parent did not receive college education was 13.0 ± 3.8 nmol/L. The concentration of 25OHD was higher in samples taken in summer (May–October) than in winter (November–April; mean difference 10.1 ± 3.5 nmol/L). A concentration of 25OHD <50 nmol/L and 25OHD between 50 and 75 nmol/L were seen in 4 (3.4%) and 34 (29.1%) infants, respectively, in summer months (May–October).

Thirteen (6.4%) infants had no vitamin D supplementation after birth and had the lowest 25OHD concentrations (67.8 ± 23.6 nmol/L) at 6 months. Most infants (*n* = 159, 78.7%) were given vitamin D supplementation ≥400 IU/day. But even in these 159 infants, 5 (3.1%) had 25OHD concentrations <50 nmol/L, and 51 (32.1%) had 25OHD concentration between 50 and 75 nmol/L. The mean difference of 25OHD between supplement user (> or = 400 IU/day) and non‐users was 18.5 ± 7.0 nmol/L (*p* = .009), and the mean difference of 25OHD between optimal supplement users (>400 IU/day) and suboptimal supplement users (<400 IU/day) was 18.5 ± 6.0 nmol/L (*p* = .003; Table 3).

**Table 3:** The frequency of different factors that may affect infant 25OHD levels and *M* ± *SD* infant serum 25OHD levels (*n* = 202)

Variable	Frequency (%)	*M* ± *SD* 25OHD at 6 months (nmol/L)	*p* value
Infant sex			.2
Male	123 (60.9)	84.8 ± 27.3	
Female	79 (39.1)	79.9 ± 20.6	
Maternal childbearing age (year)			.9
<30	98 (48.5)	82.3 ± 20.6	
> or = 30	104 (51.5)	83.4 ± 28.5	
Paternal childbearing age (year)			.8
<30	72 (35.6)	81.9 ± 20.2	
> or = 30	130 (64.4)	83.8 ± 27.8	
Maternal education			.03
College	149 (73.8)	86.1 ± 26.4	
Non‐college	53 (26.2)	76.8 ± 22.3	
Paternal education			.02
College	141 (69.8)	86.7 ± 27.3	
Non‐college	61 (30.2)	76.7 ± 19.9	
Parents education			.001
A least one not receiving third level education	80 (39.6)	75.8 ± 20.8	
Both receiving third level education	122 (60.4)	88.8 ± 27.3	
Season of sampling			.003
Summer (May–October)	117 (57.9)	87.1 ± 25.6	
Winter (November–April)	85 (42.1)	77.1 ± 22.8	
Initiated weaning at 6 months			.9
Yes	165 (81.7)	83.4 ± 26.4	
No	37 (18.3)	82.5 ± 19.7	
Feeding patterns			.8
Exclusive breastfeeding	109 (54.0)	81.8 ± 22.8	
Mixed feeding	53 (26.2)	83.4 ± 25.3	
Formula milk	40 (19.8)	85.1 ± 30.0	
Vitamin D supplementation			.001
Never supplemented	13 (6.4)	67.8 ± 23.6	
<400 IU/day	30 (14.9)	71.2 ± 17.1	
> or = 400 IU/day	159 (78.7)	86.3 ± 25.3	
Infant outdoor time			.2
> or = 1 hr/day	118 (58.4)	84.9 ± 25.7	
<1 hr/day	84 (41.6)	84 ± 23.6	

*Note*. Differences between two groups were tested using analysis of independent *t* test. Differences among three or more groups were tested using one way analysis of variance using square‐root transformed values.

Abbreviation: 25OHD, 25‐hydroxyvitamin D.

At 6 months, more than half of the infants (*n* = 109, 54.0%) were still exclusively breastfed, 53 (26.2%) were mixed‐fed (breast milk and formula milk), and 40 (19.8%) were formula‐fed (823 ± 136 ml/day). Most infants (*n* = 165, 81.7%) had already been introduced to solid food, although milk was still their main food source at the age of 6 months. There was no difference in 25OHD concentrations among infants with different feeding patterns.

The main modifiers (adjusted estimate [95% CI]; nmol/L) for 25OHD at 6 months included season (9.7 [2.7, 16.8], *p* = .007), dosage of vitamin D supplementation (12.3 [6.1, 18.4], *p* < .001), parental education (10.9 [3.6, 18.2], *p* = .004), and feeding patterns (4.7 [0.2, 9.1], *p* = .04), accounting for serum 25OHD at birth with overall adjusted *r* ^2^ = 0.187 in the whole model (Table 4). We found formula‐fed infants had a greater increase in serum 25OHD concentrations from birth to 6 months than breastfed infants with mean difference of 12.9 ± 5.2 nmol/L (*p* = .01) and also than mixed‐fed infants with mean difference of 11.7 ± 5.9 nmol/L (*p* = .05).

**Table 4:** Modifiers of serum 25OHD (nmol/L) at 6 months (*n* = 202) using a linear regression model

Predictor	25OHD Mean (*SD*)	Adjusted estimate (95% CI)	*p* value
Season of blood sample			.007
Winter (November–April; *n* = 85)	77.1 ± 22.8	Reference	
Summer (May–October; *n* = 117)	87.1 ± 25.6	9.7 (2.7, 16.8)	
Infants receiving vitamin D supplements			<.001
Never (*n* = 13)	67.8 ± 23.6	Reference	
<400 IU/day (*n* = 30)	71.2 ± 17.1		
> or = 400 IU/day (*n* = 159)	86.3 ± 25.3	12.3 (6.1, 18.4)	
Parental education			.004
A least one did not receive third level education	73.7 ± 20.0	Reference	
Both received third level education	88.8 ± 27.3	10.9 (3.6, 18.2)	
Cord blood 25OHD (nmol/L)	continues	0.14 (‐0.1, 0.4)	.2
Feeding patterns			.04
Exclusive breastfed	81.8 ± 22.8	Reference	
Mixed‐fed	83.4 ± 25.3		
Formula‐fed	85.1 ± 30.0	4.7 (0.2, 9.1)	

Abbreviation: 25OHD, 25‐hydroxyvitamin D; CI, confidence interval.

### 3.3 Selected case discussion

In the current study, the 25OHD concentration of 11 infants was <50 nmol/L at 6 months of age (Table 5): three of them never used vitamin D supplementation, another three were supplemented with less than the recommended dosage (100–200 IU/day on average), and the other five infants were supplemented with the recommended dose of vitamin D (> or = 400 IU/day), but for four of these infants, blood sampling occurred in winter. Only one breastfed subject who was supplemented with recommended dosage of vitamin D (400 IU/day) and sampled during summer had serum 25OHD 44.8 nmol/L, although this case had a sufficient concentration at birth (86.1 nmol/L).

**Table 5:** Individual case information on infants with low serum 25OHD levels (<50 nmol/L) at 6 months (*n* = 11)

Case (No.)	Cord 25OHD (nmol/L)	25OHD at 6 months (nmol/L)	Maternal vitamin D supplementation during pregnancy (IU/day)	Vitamin D supplementation after birth (IU/day)	Season of blood sample	Parental education	Feeding pattern
Case 588	76.4	24.5	125	400	Winter	Both college	Breastfed
Case 458	72.6	30.3	1666	0	Winter	One college	Breastfed
Case 731	23.2	34.9	620	500	Winter	One college	Formula‐fed
Case 207	35.8	43.2	500	0	Summer	Both college	Breastfed
Case 361	47.7	44.5	0	0	Summer	One college	Breastfed
Case 106	86.1	44.8	383	400	Summer	Both college	Breastfed
Case 659	20.4	47.4	400	200	Winter	Both college	Mixed‐fed
Case 666	33.6	47.5	500	400	Winter	One college	Breastfed
Case 761	49.3	47.9	500	400	Winter	Both college	Mixed‐fed
Case 613	31.6	48.3	0	150	Winter	One college	Mixed‐fed
Case 24	41.9	48.5	625	100	Summer	None college	Formula‐fed

Abbreviation: 25OHD, 25‐hydroxyvitamin D.

In the current study, there were 13 infants who never received vitamin D supplementation during their first 6 months of life (Table 6). Three of them had serum 25OHD concentrations < 50 nmol/L at 6 months, and they were all exclusively breastfed. Four infants had 25OHD >75 nmol/L; three of them being formula‐fed and one mixed‐fed. The other three cases that had 6‐month blood sampled during the summer months all had low 25OHD at birth (26.0, 27.9, and 28.9 nmol/L). In addition, case 711 was exclusively breastfed also had reasonable concentration of 25OHD (71.6 nmol/L) at 6 months even without infantile supplementation, with maternal vitamin D supplementation of 1,000 IU/day during pregnancy and with 25OHD concentration 70 nmol/L in cord blood.

**Table 6:** Individual case information on infants never given vitamin D supplementation during first 6 months of life (*n* = 13)

Case (No.)	Cord 25OHD (nmol/L)	25OHD at 6 month (nmol/L)	Maternal vitamin D supplementation during pregnancy (IU/day)	Vitamin D supplementation after birth (IU/day)	Season of blood sample	Parental education	Feeding pattern
Case 485	72.6	30.3	1666	0	Winter	One college	Breastfed
Case 207	35.8	43.2	500	0	Summer	Both college	Breastfed
Case 361	47.7	44.5	0	0	Summer	One college	Breastfed
Case 26	24.5	54	500	0	Summer	One college	Formula‐fed
Case 199	41.7	62.1	500	0	Summer	Both college	Formula‐fed
Case 242	52.1	63.9	600	0	Summer	Both college	Mixed‐fed
Case 487	24	69.1	450	0	Winter	Both college	Mixed‐fed
Case 711	70	71.6	1000	0	winter	Both college	Breast fed
Case 355	27	72.6	833	0	Summer	Both college	Formula‐fed
Case 375	26.0	78.4	0	0	Summer	None college	Formula‐fed
Case 310	27.9	81	800	0	Summer	None college	Formula‐fed
Case 750	70.4	87	1500	0	Winter	Both college	Formula‐fed
Case 420	28.9	124.2	400	0	Summer	Both college	Mixed‐fed

## 4 DISCUSSION

This is a longitudinal follow‐up study carried out in Guangzhou, China (23^o^N), investigating the 25OHD levels in infants at birth (cord blood) and at 6 months of age, taking consideration of both maternal and infant vitamin D supplementation, infant feeding patterns, and seasonal effects. A study carried out among 148 4‐month‐old infants in Izmir, Turkey (38°N) reported the *M* ± *SD* 25OHD was 75.2 ± 27.3 nmol/L (Halicioglu et al., 2012). The infants' ages were similar to the current study, but they were exclusively breastfed and had lower 25OHD levels than reported here. Another study performed in Aalborg, Denmark (*n* = 90, age = 4 months, 57°N) reported the *M* ± *SD* 25OHD was 94.1 ± 24.2 nmol/L (Streym et al., 2016). The infants' age were also similar with our study, but this study focused on vitamin D content in human breast milk and did not describe the details of the infants. A study carried out among 76 infants at the age of 12 months in Iceland (63–66°N) reported the *M* ± *SD* 25OHD was 98.1 ± 32.2 nmol/L (Thorisdottir, Gunnarsdottir, Steingrimsdottir, Palsson, & Thorsdottir, 2014). This level seems higher than in the current study, but the age of the infants is older, and they may have more opportunities for outdoor activity and obtain vitamin D from many fortified foods, such as formula milk, porridge, and breakfast cereals, which have been fortified in Iceland since 2003 (Thorisdottir, Thorsdottir, & Palsson, 2011). The cohort presented in the current study is much larger than these previous studies and included infants with different feeding patterns, and the blood sampling was across the calendar year. A large population‐based study performed in Wuxi, China (32°N; Zhao et al., 2015) reported serum 25OHD concentrations in 3,845 1‐year‐old children with median (IQR) of 74.7 (56.4, 94.3) nmol/L. Our study found the *M* ± *SD* 25OHD was 82.9 ± 24.9 nmol/L and median (IQR) 25OHD was 80.8 (67.5, 95.7) nmol/L in 6‐month‐old infants. The vitamin D status reported in our study appears to be higher than reported in the Wuxi study and may reflect the lower latitude of Guangzhou. Based on the guidelines of the Endocrine Society clinical practice (Holick, Binkley, & Bischoff‐Ferrari, 2011) and Institute of Medicine (2011), we used 30, 50, and 75 nmol/L as cutoffs for 25OHD deficiency, insufficiency, and overall health, respectively. Serum 25OHD concentrations at 6 months <30 nmol/L was only seen in one participant, and concentrations between 30 and 50 nmol/L were reported in 10 (5.0%) subjects. Although the majority of infants (*n* = 120, 59.4%) had 25OHD >75 nmol/L at 6 months, more than one‐third of infants still did not reach 75 nmol/L. Cord serum 25OHD levels at birth were much lower than the levels at 6 months. The only source of vitamin D for the foetus is maternal transfer. Maternal vitamin D supplementation and sun exposure during pregnancy could increase cord 25OHD, but based on data from our study, neither maternal supplementation nor outdoor activity during pregnancy reached sufficient levels to prevent the newborns from vitamin D deficiency. After birth, infants can get vitamin D in many ways including through skin synthesis, diet, and vitamin D supplementation. In the current study, 93.6% infants were supplemented with vitamin D during the first 6 months of life, which was the main factor boosting 25OHD levels 6 months compared with levels at birth. The results show a much higher level of parental adherence to the Chinese medical recommendation of vitamin D supplementation after birth than many other regions of the world (Uday, Kongjonaj, Aguiar, Tulchinsky, & Högler, 2017). First, with the rapid development of China's economy, parents' awareness of children's healthcare has been greatly enhanced. Second, all the subjects in the study were from a first‐tier city (Guangzhou) in China, where the community medical level is relatively high. Parents can get relevant information from medical staff during pregnancy, delivery, and postpartum. Third, parents of the subjects in this study were highly educated, with greater than 50% of the parents receiving tertiary education.

In this study, we found infants born to higher educated parents had higher serum 25OHD levels at 6 months. Parents with higher education had more knowledge and paid more attention to nutrition in general. Guangzhou is located on the subtropical China coast and has a maritime subtropical monsoon climate. The annual average temperature is 20–22°C, with minimal annual fluctuations in temperature and day length. The hottest month of the year is July, with an average monthly temperature of 28.7°C, and the coldest month is January, with an average monthly temperature is 9–16°C. The average relative humidity is 77%, and the annual rainfall is about 1,720 mm. In 2018, Guangzhou had 165 raining days, 42 sunny days, and 149 cloudy days. We defined May–October as summer and November–April as winter for the small annual average temperature difference. Despite the abundance of sunlight in Guangzhou and high compliance of infantile vitamin D supplementation, there are still one‐third of infants with 25OHD less than 75 nmol/L at 6 months even during summer months (May–October). The reasons might be limited outdoor activity and a tendency to be covered up when going outdoors. We found formula‐fed infants had a greater increase in serum 25OHD concentrations from birth to 6 months than breastfed infants and mixed‐fed, and feeding patterns was a predictor that could influence 25OHD levels at 6 months after considering the effects of cord blood 25OHD in the regression models, due to formula milk normally being supplemented with vitamin D. Therefore, vitamin D supplementation is particularly important for breastfed infants, and they might require high dosage of vitamin D supplementation.

Although the Chinese Medical Association recommends that all children must receive 400 IU/day of vitamin D until the age of 2 years (*Chinese Journal of Pediatrics.,* 2008), there were still 13 (6.4%) infants who did not receive vitamin D supplementation, and 30 (14.9%) infants were given an insufficient dose of vitamin D supplementation (<400 IU/day). Most infants (*n* = 159, 78.7%) were given vitamin D supplementation of >400 IU/day. But in these 159 infants, there were still 5 (3.1%) with 25OHD concentrations <50 nmol/L and 51 (32.1%) with 25OHD concentrations between 50 and 75 nmol/L. First, not all infants were supplemented according to the guidelines, and the implementation of infant supplementation during the first year of life needs to be improved. Second, even among the infants who were supplemented according to current guidelines in China, there are still one‐third of the infants not reaching the optimal 25OHD concentrations (>75 nmol/L). It is possible that some parents were not fully truthful in completing the questionnaires regarding the amount of vitamin D supplementation their babies were receiving, although we believe this phenomenon is rare. A double‐blind, randomized clinical trial carried out in Canada (53^o^N) among 132 1‐month‐old healthy, term, breastfed infants who were randomly assigned to four groups receiving different dosages of vitamin D supplementation (400, 800, 1,200, and 1600 IU/day) and were followed up to 11 months. Just over half of the infants in the 400 IU/day group achieved serum 25OHD concentrations of >75 nmol/L versus 81% in the 800 IU/day group, 92% in the 1,200 IU/day group, and 100% in the 1,600 IU/day group at 3 months. However, the dosage of 1,600 IU/day has been associated with hypercalcemia (Gallo et al., 2013). More dose‐response studies are needed to define a suitable dosage of vitamin D supplementation during infancy.

The vitamin D concentration of human breast milk is very low if the mother is vitamin D deficient. A recent study carried out among 107 exclusively breastfeeding infants from 2 weeks after birth to 9 months in Denmark found median (IQR) daily intake through breast milk of vitamin D was only 77 IU/day (52–110 IU/day; Streym et al., 2016). Although a recent randomized, controlled trial found that maternal vitamin D3 supplementation alone with 6,000 IU/day for 6 months achieved similar adequate vitamin D status as in infants on direct vitamin D_3_ supplementation of 400 IU/day plus maternal 600 IU/day supplementation and safely optimized maternal vitamin D status with increase in milk vitamin D content (Dawodu, Salameh, Al‐Janahi, Bener, & Elkum, 2019). But vitamin D supplements during lactation have not received enough attention in China. In this study, maternal use of vitamin D supplementation during lactation is only 58.6% with the dose of 500 (200, 833) IU/day, which is much smaller than 6,000 IU/day. Although most infants (81.7%) in this study had to add a small amount of assisted food, milk was still their main source of nutrients. Formula‐fed infants' formula milk supply was 823 ± 136 ml/day on average in this study. At the same time, there are limited natural foods that contain high levels of vitamin D and vitamin D food fortification is lacking in China, so vitamin D from assisted food is very limited.

This study provided a unique insight of individual nutritional case reports on selected individuals. There were 11 infants who had 25OHD concentrations <50 nmol/L at 6 months in this study, and only one infant had 25OHD concentration of 24.5 nmol/L. This infant had relatively high cord 25OHD (76.4 nmol/L) and used vitamin D supplementation 400 IU/day after birth, but the levels of 25OHD at 6 months were very low. Obviously, 400 IU/day vitamin D supplementation did not prevent severe vitamin D deficiency in this individual, which suggested a personalized supplementation plan might be needed in some individuals. We also found another four infants who used sufficient vitamin D supplementation yet still had 25OHD <50 nmol/L. Except for the influence of season and feeding patterns, there was a special case that had low serum 25OHD both at birth (23.2 nmol/L) and 6 months (34.9 nmol/L) even with regular vitamin D supplementation during pregnancy (620 IU/day) and after birth (500 IU/day). In contrast, we found four infants who never used vitamin D supplementation but had high 25OHD concentration (>75 nmol/L) at 6 months, maybe due to season and feeding patterns. In addition, an exclusively breast‐fed infant also had a reasonable concentration of 25OHD (71.6 nmol/L) even without infantile supplementation, with maternal vitamin D supplementation of 1,000 IU/day during pregnancy. Maternal vitamin D supplementation still plays a certain role in promoting infants vitamin D status after birth.

Some studies have found genetic variants in relation to vitamin D synthesis, metabolism, and transportation (Kuhn et al., 2014; Suaini et al., 2014). Several twin studies have suggested that the vitamin D status as measured by serum concentrations of 25OHD is strongly (>50%) defined by genes (Jameson & De Groot, 2016). A polymorphism in the gene encoding vitamin D‐binding protein is the main driver of known genetic variation in levels of 25OHD (Wang et al., 2010; Bouillon, 2010). Individualized nutrition in relation to vitamin D supplementation might be needed in some individuals depending on their genetic variants. Vitamin D binding protein (DBP) occurs as three common allelic forms (Gc1F, Gc1S, and Gc2) whose frequencies greatly differ by race/ethnicity (Braun, Bichlmaier, & Cleve, 1992). A recent study analysed the association of vitamin D intake to circulating 25OHD concentration according to DBP genetic diversity in a group of 123 children who were identified as African–American, Hispanic, or non‐Hispanic Caucasian. They found in children meeting the U.S. recommended daily allowance of vitamin D intake (currently 600 IU/day), and the presence of the 1S DBP form (common with European ancestry) was associated with significantly higher plasma 25OHD concentrations. In the children of African ancestry attaining vitamin D recommended daily allowance, mean vitamin D sufficiency was only reached in the minority who carried the 1S DBP allele (Newton et al., 2019). Although the subjects of the current study are of the same ethnic background (Han nationality) and all of them were settled and raising their babies in Guangzhou, their ancestral homes may be different, and the genetic factors affecting vitamin D metabolism may be different. Further study is required to determine whether genetic variations affect vitamin D status in Chinese populations.

## 5 CONCLUSIONS

Serum 25OHD levels were low at birth in a southern Chinese population. Infantile supplementation is an effective way to improve 25OHD status, measured at 6 months. The current Chinese vitamin D supplementation guidelines of 400 IU/day only meets the needs of two‐thirds of infants at 6 months in our cohort using 75 nmol/L as a cutoff for overall health. Dose‐response, randomized control studies are needed to clarify the optimal amount of vitamin D supplementation during infancy in China. Exclusively breastfed infants might need greater levels of vitamin D supplementation, and individualized vitamin D supplementation plans might be needed.

## CONFLICTS OF INTEREST

The authors declare that they have no conflicts of interest.

## CONTRIBUTIONS

JW designed the study, was responsible for data collection, conducted the data analysis and drafted the initial manuscript. JYZ contributed to the design of the study, was responsible for bio‐banking, and revised the manuscript. RW was responsible for data collection, and SPH was responsible for the sample collection. TL was responsible for sample analysis. MZT and GEL contributed to the design of the study, supervised the overall project, reviewed and revised the manuscript, and were responsible for the final content of the manuscript.
